# Pollen allergen skin test and specific IgE reactivity among Filipinos: a community-based study

**DOI:** 10.1186/s13223-020-00471-9

**Published:** 2020-08-12

**Authors:** Maureen Sabit, Cecil Wong, Agnes Andaya, John Donnie Ramos

**Affiliations:** 1 The Graduate School, University of Santo Tomas, 1008 Manila, Philippines; 2 Research Center for the Natural and Applied Sciences, Thomas Aquinas Research Complex, University of Santo Tomas, 1008 Manila, Philippines; 3 Department of Biological Sciences, College of Science, University of Santo Tomas, 1008 Manila, Philippines; 4 Department of Pediatrics, Section of Allergy and Clinical Immunology, University of Santo Tomas Hospital, 1008 Manila, Philippines

**Keywords:** Pollen allergen, Enzyme-Linked Immunosorbent Assay, Skin prick tests, Sensitization

## Abstract

**Background:**

Despite the clinical importance of pollen allergens among Filipinos, few studies delve into the sensitization profiles of Filipinos against pollen allergens. This study determined the sensitization profile of Filipinos to pollen using skin prick test (SPT) and pollen-specific ELISA.

**Methods:**

Pollen from fifteen selected plant sources was collected and extracted for use in sensitization tests. Volunteers were interviewed for their clinical history prior to blood sampling and SPT. The blood samples collected were assessed using Enzyme-Linked Immunosorbent Assay (ELISA).

**Results:**

The best panel of pollen allergens for the skin prick test was *Mangifera indica* (64%), *Acacia auriculiformis* (28%), *Mimosa* spp. (25%) *Amaranthus spinosus* (22%), *Lantana camara* (20%), *Pilea microphylla* (16%) and *Dichanthium aristatum* (15%). Young adults had more sensitizations to pollen than among early childhood and elderly. There were more allergic subjects that have rhinitis (61%) than asthma (42%) and atopic dermatitis (35%). Pollen-specific IgE levels show low percent reactivity as compared to the skin test with *Cocos nucifera* obtaining the highest IgE reactivity (21%).

**Conclusions:**

Pollen allergens from both arboreal and herbaceous plants used in this study yielded positive reactivities for both skin tests and specific IgE tests.

## Background

Allergy is a hypersensitive reaction characterized by an immune-mediated inflammatory response to common environmental protein allergens that are deemed to be harmless in non-allergic individuals [1]. The global increase in the prevalence of allergic respiratory diseases and their effect on the quality of life of allergic patients is a health issue that needs immediate attention [2, 3]. In the Philippines, the reported overall prevalence of allergic rhinitis and allergic rhinoconjunctivitis is 20% and 14%, respectively [4]; whereas, work absence due to asthma is reported at 46.6% [5]. Pollen is one of the most common and important sensitizing aeroallergens [6–9] that cause respiratory allergies such as allergic rhinitis, allergic asthma, and atopic dermatitis. Dissemination or dispersal of pollen, which occurs during a plant’s pollination or flowering period, ensures survival and continuity of its lineage. Small, lightweight pollen, which is produced in copious amounts by anemophilous (wind-pollinated) plants, are the major allergens in the atmosphere. Several studies have shown that the incidence of pollinosis in urban areas is higher than the countryside due to unsuitable green space construction, urban heat island effect, and traffic pollution [10].

In tropical Asia, little information on pollen allergens is available [11], and information on the sensitization profiles of Filipino allergic patients to pollen allergens is limited. Sensitization to grass and weed pollen among Filipinos was reported earlier [12] and described anew by recent studies using immunobiological techniques [13, 14]. It is in this context that this study was conducted. Aqueous extracts of tree and weed pollen found to be abundant in the atmosphere were used to generate the sensitization profile of Filipinos using skin tests. This study aims to determine the total IgE and pollen specific-IgE profiles of skin-test positive and negative subjects and correlate skin tests with pollen-specific IgE reactivity.

## Materials and methods

### Study area and subjects

Vegetation or the “*green space*” in a highly urbanized city of Metro Manila, is found only in parks, gardens, and trees planted along the road. Ten barangays near the vicinity of a pollen trap (situated within the University of Santo Tomas, Manila) were randomly chosen for this study. Prior to sampling, an approval from the UST Graduate School Institutional Ethics Committee Review Board was obtained. A total of 541 volunteers who have been living, working, or studying in the “*University Belt*” for more than 2 years prior to the conduct of this study were recruited. SPT-positive subjects were designated as cases while SPT-negative subjects were controls. All participants gave their informed consent prior to answering a standardized questionnaire. This questionnaire was adapted and modified by De Guia [15] from previous sources [16–18], and, validated these questionnaires for Filipino patients.

### Pollen collection

The fifteen plants chosen as sources of pollen were previously reported as widespread in Metro Manila, and most are representative of the plant families with a high prevalence of airborne pollen [19]. Included were arboreal plants: *Ptychosperma macarthurii* (PTY), *Cocos nucifera* (COC), *Mangifera indica* (MAN), *Acacia auriculiformis* (ACA), *Senna siamea* (SEN), *Lantana camara* (LAN), *Carica papaya* (CAR), *Terminalia catappa* (TER), *Eucalyptus* spp. (EUC), *Pinus kesiya* (PIN), *Dendrocnide meyeniana* (DEN); and herbaceous plants: *Mimosa* spp. (MIM), *Dichanthium aristatum* (DIC), *Pilea microphylla* (PIL), and *Amaranthus spinosus* (AMA). Pollen samples were collected from the mature anthers of these plants and processed as described [20]. Flowers from trees and weeds were dried then passed through reducing sizes of mesh sieves (150, 75, 50 and 25 µm, respectively). The presence of pollen was confirmed under a stereomicroscope (BS-2030T Digital Biological Trinocular Microscope). Pollen was stored in a tightly sealed container with a desiccant at 4 °C.

### Pollen extraction, protein assay, and preparation of pollen extracts

One gram (dry weight) of pollen was mixed with 10 mL diethyl ether and placed on a shaker overnight. The defatted pollen was centrifuged at 4000 rpm for 10 min, left to dry overnight, and mixed with 10 mL Phosphate Buffered Saline (PBS). The mixture was stirred overnight at 4 °C and centrifuged at 13,000 rpm, 4 °C for 30 min. The supernatant was transferred to a dialysis tubing (6–8 kD MWCO, supplied by Spectrum Labs, USA) and passed through a 0.2 μm Millipore filter (Whatman Puradisc 25, PES sterile). 1 mL aliquots of the dialyzed products were transferred to microcentrifuge tubes and stored at − 20 °C until use. The total protein content of the pollen was analyzed using Bio-Rad Protein Assay Kit II (Bio-Rad Laboratories Inc, Hercules, CA, USA). Each crude pollen aqueous extract was diluted with the appropriate amount of PBS to get a final concentration of 10 µg/mL for use in skin prick test (SPT) and pollen-specific IgE ELISA.

### Skin-prick test

Volunteer selection was made carefully following the guidelines as described [1]. SPT was performed on all participants using a panel of 15 crude pollen extracts, house dust mites (HDM) *Suidaisia pontifica* (SUD), and *Blomia tropicalis* (BLO), and with histamine (0.1%) in PBS and physiologic saline solution as positive and negative controls, respectively. A drop of each pollen extract was directly pricked on the participant’s forearm using a 1 mm-point sterile lancet. A white wheal measuring ≥ 3 mm in diameter and a red flare around the pricked skin area was interpreted as positive SPT [21].

### Immunobiological methods-Enzyme-Linked Immunosorbent Assay (ELISA)

Five mL peripheral blood of study participants was collected in EDTA tubes and then centrifuged to separate the plasma. Samples were aliquoted and stored at − 20 °C until further use. The total IgE levels of cases (n = 130) and controls (n = 110) were determined following the manufacturer’s protocol (Human IgE ELISA Core Kit, Komabiotech, South Korea). For pollen-specific IgE ELISA, 10 μg/mL aqueous pollen extracts diluted in carbonate-bicarbonate buffer were coated overnight at 4 °C onto the wells of high-binding microtiter plates (Corning Costar, NY, USA). Plates were blocked with 1% BSA (Sigma-Aldrich, Saint Louis, MO, USA) in PBS for 1 h at room temperature. Plasma samples from both cases and controls were dispensed in duplicates onto the wells and incubated for 1 h at room temperature. Plates were incubated with 500× dilution of an HRP-conjugated goat anti-human IgE for 1 h at room temperature. Colorimetric reactions for all immunobiological tests were performed using TMB (3,3′,5,5′-Tetramethylbenzidine) and the reaction was stopped with 2 M H_2_SO_4_. Absorbance was read at 450 nm using Bio-Tek ELX800 ELISA reader (Tecan, Austria).

### Statistical analysis

Data characteristics of test subjects (cases and controls) and positive reactions to different pollen allergens using skin prick test and specific IgE ELISA were presented as frequency (percentage) and compared using the Chi square test of homogeneity or Fisher’s Exact test or z-test for two sample proportions. The diagnostic performance of SPT (positive and negative predictive values, specificity and sensitivity) with pollen, was computed. Spearman’s correlation coefficient was used to test the association of the different pollen based on pollen-specific IgE ELISA results. Percent reactivity of pollen-specific IgE and sensitization profile among age groups were graphically represented. Frequency tables and graphs were created, and data analyzed using SPSS (v.20), MS Excel and/or GraphPad Prism 8.

## Results

Five hundred forty-one study subjects from Metro Manila were recruited. Seven percent (7%) of the study subjects were excluded because of non-cooperation, had taken antihistamine drugs before testing and backing out at the last minute. Of the 41% that were positive to skin tests (n = 205), 49% (n = 101) were positive to both pollen and HDM, 14% (n = 29) were positive only to pollen, and 37% (n = 75) were positive only to HDM. Subjects that tested positive to pollen (cases, n = 130), who self-reported to having allergic asthma (AA), allergic rhinitis (AR), and atopic dermatitis (AD), were referred to as allergic (79%), while those who self-reported not to have allergic diseases were asymptomatic. Of the 373 skin-test negative subjects (to pollen), 110 were asymptomatic and referred to as controls. Table 1 shows the characteristics of the test subjects (cases and controls). Significant differences were shown in the percentage number of children (2–9 years old), young adults (20–40 years old), between gender and those with a family history of allergies between cases and controls. Of asymptomatic cases (n = 27), 37% have family members that were allergic. Allergic asthma (AA) and AR were commonly reported in either the father or mother of the cases. Several cases and controls have pets at home, and there were no significant differences between them. The most common household pet were dogs (cases—68%, controls—88%), cats and birds (cases—10%, controls—12%) and a combination of dogs, cats, and birds (for cases only—22%). Likewise, no significant differences were shown between cases and controls who self-reported to smoke or have family members who smoke. Allergic rhinitis (61%) was prevalent among allergic cases, followed by AA (42%) and AD (35%).

**Table 1:** Demographic characteristics of the respondents (N = 240)

Characteristic	Cases (n = 130)	Controls (n = 110)	Statistic	*p*-value (two-tailed)
Age group (years old)				
2–9	8 (6.15%)	28 (25.45%)	− 4.17^b^	0.001
10–19	29 (22.31%)	21 (19.09%)	0.61	0.541
20–40	67 (51.54%)	42 (38.18%)	2.07^a^	0.038
41–60	23 (17.69%)	13 (11.82%)	1.27	0.205
≥ 61	3 (2.31%)	6 (5.45%)	− 1.28	0.202
Sex			3.88^a^	0.049
Male	60 (46.15%)	37 (33.64%)		
Female	70 (53.85%)	73 (66.36%)		
Family history of allergies			13.97^b^	0.001
Yes	65 (50.00%)	29 (26.36%)		
No	65 (50.00%)	81 (73.64%)		
Presence of pets at home			3.68	0.055
Yes	81 (62.31%)	55 (50.00%)		
No	49 (37.69%)	55 (50.00%)		
Smokes cigar, tobacco, or pipe			2.78	0.095
Yes	64 (49.23%)	66 (60.00%)		
No	66 (50.77%)	44 (40.00%)		

Data are presented as frequency (percentage) unless otherwise stated. Comparative analysis was conducted using the Chi Square Test of Homogeneity, Fisher’s Exact test, or z-test for two sample proportions

^a^Significant at 0.05

^b^Significant at 0.01

Total IgE values of ≥ 100 IU/mL of subjects in cases and controls were 87% (n = 113) and 55% (n = 60), respectively. Based on Spearman’s rho, there was no significant difference in the mean IgE levels between gender (cases and controls) and presence of allergic diseases in cases, although, the highest mean was obtained from male allergic subjects (284.58 IU/mL) and those with AA (344.82 IU/mL) and AD (324.89 IU/mL).

**Fig. 1:**
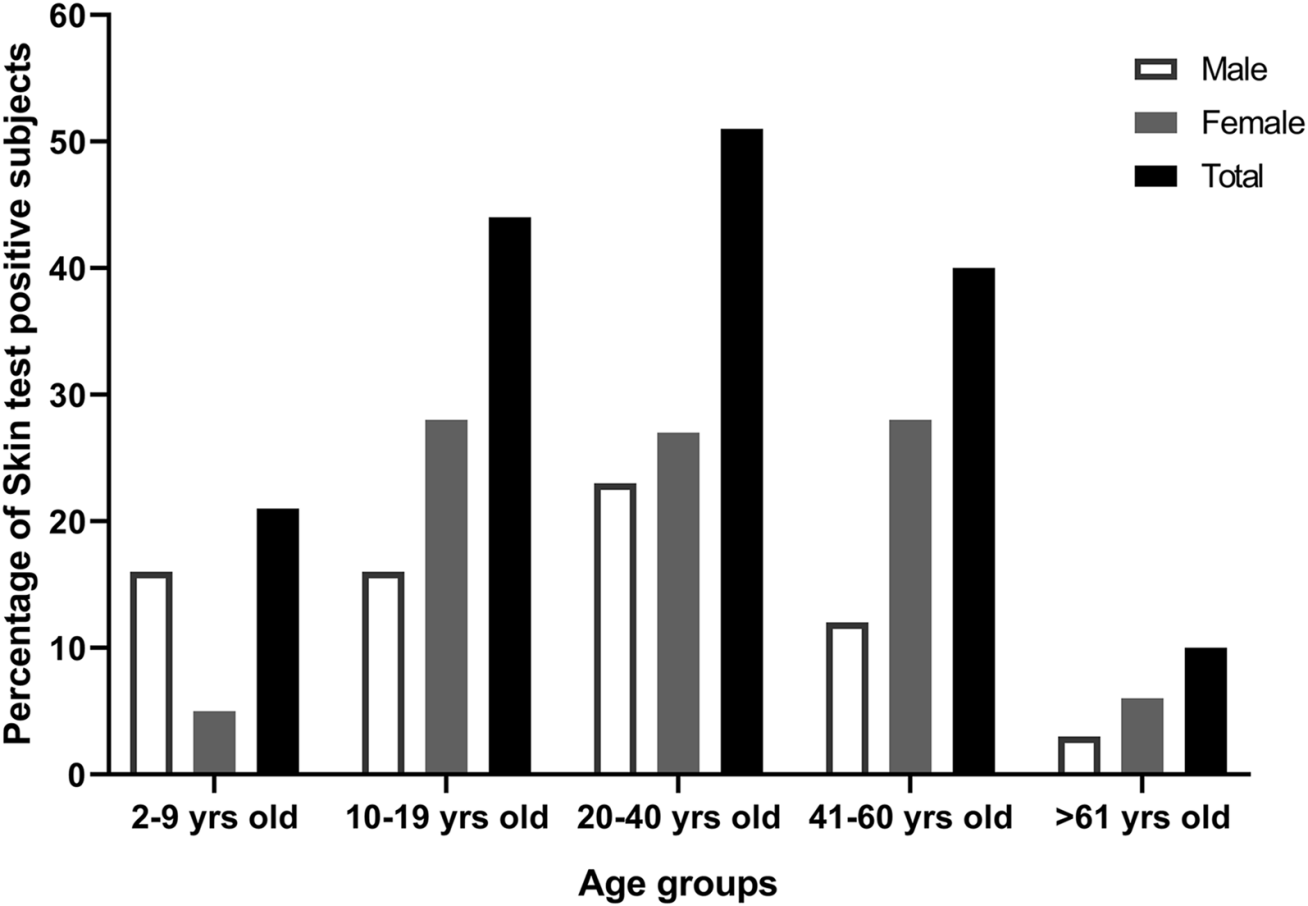
Sensitization profile of allergic subjects across age groups: childhood (2–9 years old), adolescence (10–19 years old), young adults (20–40 years old), middle-aged adults (41–60 years old) and elderly (61 years old and above)

**Fig. 2:**
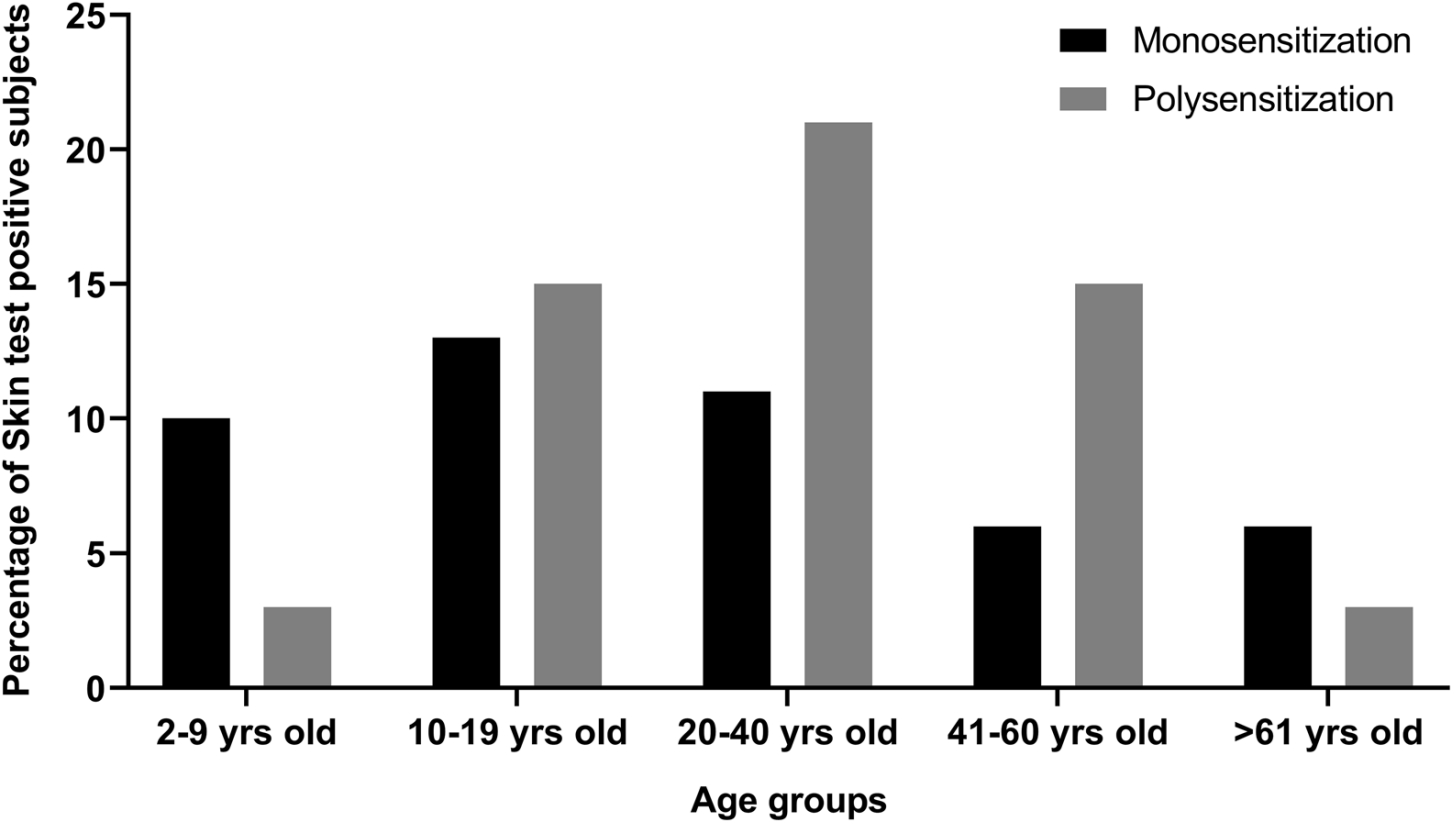
Sensitization profile of allergic subjects across age groups: childhood (2–9 years old), adolescence (10–19 years old), young adults (20–40 years old), middle-aged adults (41–60 years old) and elderly (61 years old and above)

Of the 11 species of arboreal plants used for SPT, most of the study subjects tested positive to three pollen sources: *Mangifera indica* (MAN), *Acacia auriculiformis* (ACA), and *Lantana camara* (LAN) as shown in Table 2. Study subjects were also positive to the pollen of herbaceous species, namely, *Mimosa* spp. (MIM), *Amaranthus spinosus* (AMA), *Pilea microphylla* (PIL), and *Dichanthium aristatum* (DIC). Wheal diameters of 6-10 mm were observed in subjects who tested positive to MIM and MAN. Based on sensitizations, 40% and 31% of positive subjects from skin tests and pollen-specific IgE ELISA, respectively, were sensitized to one allergen (monosensitization) while the rest of the test subjects were sensitized to two or more allergens (polysensitization). Figures 1 and 2 show the sensitization profile of allergic subjects across age groups. Male children (2–9 years old) have early sensitization than females. However, females show high sensitizations than males in all other age groups. Overall, there was an increase in the number of sensitizations to young adults (20–40 years old) and then gradually declined in the older age groups. Likewise, the number of polysensitized (65% males, 56% females) subjects show similar tendencies while monosensitization (35% male, 44% female) were more evident in younger age groups. The sensitivity and specificity of the skin tests were 30% (95% CI 25–35%) and 83% (95% CI 75–88%), respectively. The prevalence of the allergic disease in the population was 69% with a positive predictive value of 79% (95% CI 71–85%), and a negative predictive value of 34% (95% CI 30–39%).

**Table 2:** Frequency of positive reactions to allergen among cases using skin prick test and specific IgE ELISA Test (N = 130)

Sources of pollen				Frequency (Percentage)		Statistic	*p*-value (two-tailed)
Family	Scientific name	Code	Local Name (English; Filipino)	Skin Prick Test (N = 130)	Specific IgE ELISA (N = 130)		
Arboreal plants							
Anacardiaceae	*Mangifera indica*	MAN	Mango; Manga	83 (63.85%)	3 (2.31%)	10.53^b^	0.000
Arecaceae	*Cocos nucifera*	COC	Coconut; Niyog	6 (4.62%)	27 (20.77%)	− 3.91^b^	0.000
Arecaceae	*Ptychosperma macarthurrii*	PTY	MacArthurs’s palm	7 (5.38%)	20 (15.38%)	− 2.67^a^	0.008
Fabaceae	*Acacia auriculiformis*	ACA	Japanese acacia	36 (27.69%)	5 (3.85%)	5.27^b^	0.000
Fabaceae	*Senna siamea*	SEN	Thailand acacia	5 (3.85%)	10 (7.69%)	− 1.33	0.184
Caricaceae	*Carica papaya*	CAR	Melon tree; Papaya	12 (9.23%)	9 (6.92%)	0.681	0.495
Combretaceae	*Terminalia catappa*	TER	Tropical almond; talisai	4 (3.08%)	3 (2.31%)	0.382	0.702
Myrtaceae	*Eucalyptus* spp.	EUC	Eucalyptus	9 (6.92%)	15 (11.54%)	− 1.28	0.199
Pinaceae	*Pinus kesiya*	PIN	Benguet pine; Sahing	10 (7.69%)	25 (19.23%)	− 2.72^a^	0.006
Urticaceae	*Dendrocnide meyeniana*	DEN	Lipa; Lipang kalabaw	9 (6.92%)	12 (9.23%)	− 0.681	0.496
Verbenaceae	*Lantana camara*	LAN	Prickly lantana; Koronitas	26 (20.00%)	1 (0.77%)	5.07^b^	0.000
Herbaceous plants							
Amaranthaceae	*Amaranthus spinosus*	AMA	Thorny amaranth; Colitis	28 (21.54%)	13 (10.00%)	2.55^a^	0.011
Fabaceae	*Mimosa* spp.	MIM	Sensitive plant; Makahiya	33 (25.38%)	12 (9.23%)	3.44^b^	0.001
Poaceae	*Dichanthium aristatum*	DIC	Alabang grass; Alabang	19 (14.62%)	6 (4.61%)	2.73^a^	0.006
Urticaceae	*Pilea microphylla*	PIL	Gunpowder plant; Isang-dakot-na-bigas	21 (16.15%)	4 (3.07%)	3.57^a^	0.000

Data are presented as frequency (percentage) unless otherwise stated. Comparative analysis was conducted using the Chi Square Test of Homogeneity, Fisher’s Exact test, or z-test for two sample proportions

^a^Significant at 0.05

^b^Significant at 0.01

**Fig. 3:**
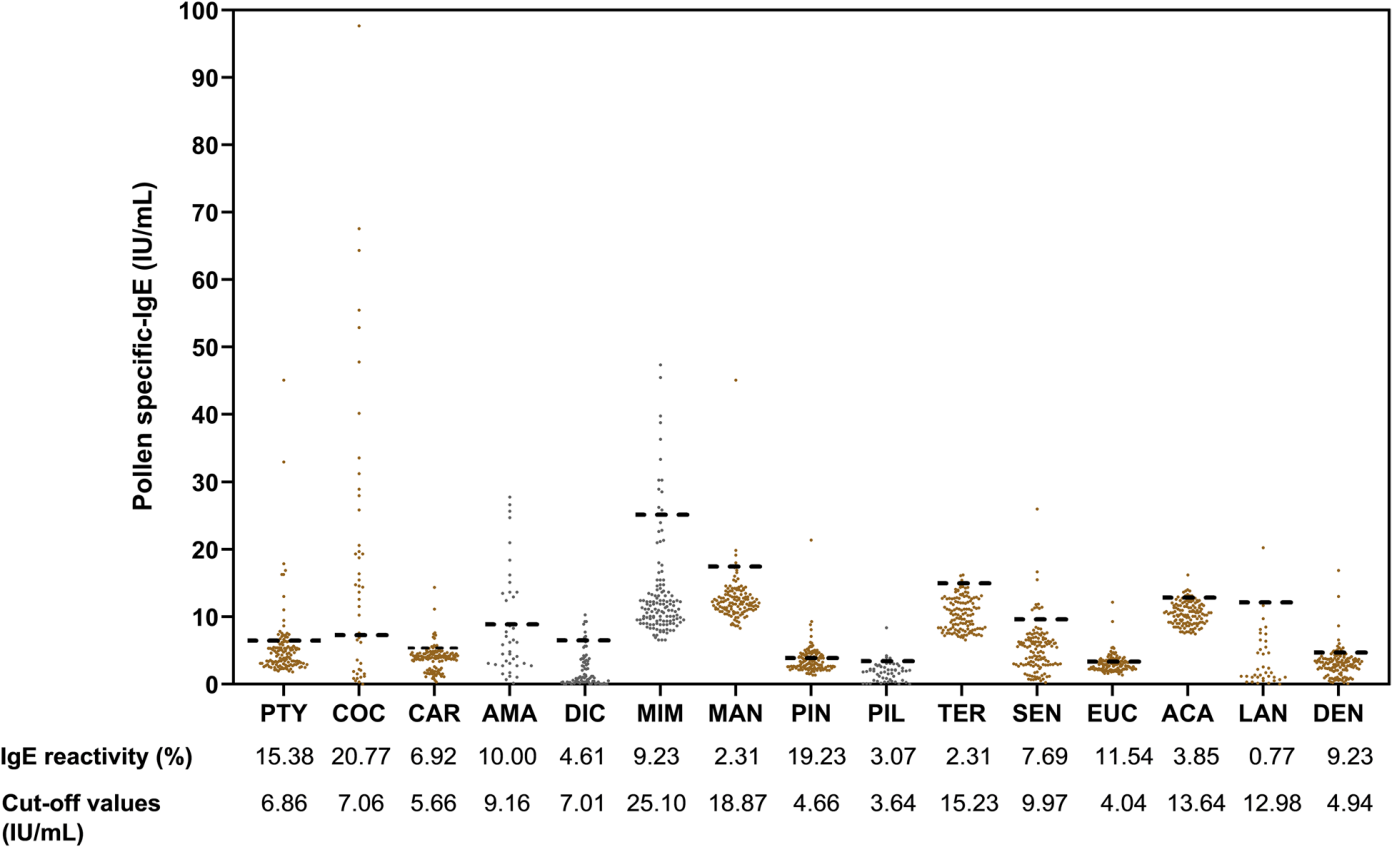
Sensitization profile of 130 Filipino allergic patients against pollen allergens (brown—arboreal plants; gray—herbaceous weeds) using specific-IgE ELISA. Broken lines denote the cut-off values computed as ${\bar{\text{X}}} \pm 2{\text{SD}}$ of 50 control subjects with Total IgE of ≤ 100 IU/mL

Significant differences in positive reaction to skin tests and pollen-specific IgE ELISA were shown in 10 pollen allergens (Table 2). Fifty-four percent (54%) of skin test positive cases were positive to pollen-specific IgE, 44% of which were asymptomatic. Subjects were more reactive to COC, PIN, and PTY among arboreal plants, and to AMA and MIM among herbaceous plants. These specific-IgE reactivities were based on cut-off values (${\bar{\text{X}}} \pm 2{\text{SD}}$) derived from 50 control subjects with a Total IgE of ≤ 100 IU/mL (Fig. 3). As shown in Table 3, MAN has significantly weak to moderate positive relationships to 11 pollen allergens. PIN, CAR, COC, EUC and ACA, PTY have weak to moderate to strong positive relationships to 10 and 9 pollen allergens, respectively. Significantly strong positive relationships were shown between EUC-PTY, EUC-PIN, PTY-PIN, and DIC-PIL. Species under the same family, e.g., Fabaceae (ACA, SEN, MIM), Arecaceae (COC, PTY), and Urticaceae (DEN, PIL) show significantly weak to moderate relationship, except for MIM that showed no relationship with ACA and SEN (Table 3).

**Table 3:** Spearman’s correlation of pollen-specific IgE (IU/mL) of different pollens (N = 130)

	MAN	ACA	MIM	PIN	CAR	AMA	COC	PIL	DEN	TER	LAN	DIC	EUC	PTY	SEN
1. MAN	- - -	- - -	- - -	- - -	- - -	- - -	- - -	- - -	- - -	- - -	- - -	- - -	- - -	- - -	- - -
2. ACA	0.42^b^	- - -	- - -	- - -	- - -	- - -	- - -	- - -	- - -	- - -	- - -	- - -	- - -	- - -	- - -
3. MIM	0.22^a^	–0.05	- - -	- - -	- - -	- - -	- - -	- - -	- - -	- - -	- - -	- - -	- - -	- - -	- - -
4. PIN	0.45^b^	0.31^b^	0.36^b^	- - -	- - -	- - -	- - -	- - -	- - -	- - -	- - -	- - -	- - -	- - -	- - -
5. CAR	0.41^b^	0.30^b^	0.15	0.38^b^	- - -	- - -	- - -	- - -	- - -	- - -	- - -	- - -	- - -	- - -	- - -
6. AMA	0.23^b^	–0.09	0.37^b^	0.26^b^	0.24^b^	- - -	- - -	- - -	- - -	- - -	- - -	- - -	- - -	- - -	- - -
7. COC	0.25^b^	0.24^b^	0.21^a^	0.27^b^	0.32^b^	0.26^b^	- - -	- - -	- - -	- - -	- - -	- - -	- - -	- - -	- - -
8. PIL	–0.08	–0.10	0.09	–0.24^b^	–0.30^b^	0.13	0.03	- - -	- - -	- - -	- - -	- - -	- - -	- - -	- - -
9. DEN	0.47^b^	0.41^b^	0.16	0.39^b^	0.29^b^	0.21^a^	0.21^a^	0.20^a^	- - -	- - -	- - -	- - -	- - -	- - -	- - -
10.TER	0.21^a^	0.44^b^	0.19^a^	0.10	0.06	0.06	0.09	0.14	0.16	- - -	- - -	- - -	- - -	- - -	- - -
11. LAN	0.14	–0.02	0.13	0.09	–0.06	0.14	0.04	0.38^b^	0.40^b^	–0.11	- - -	- - -	- - -	- - -	- - -
12. DIC	0.11	–0.12	0.29^b^	–0.04	–0.17	0.15	0.08	0.51^b^	0.16	0.10	0.46^b^	- - -	- - -	- - -	- - -
13. EUC	0.33^b^	0.22^a^	0.19^a^	0.57^b^	0.22^a^	0.19^a^	0.19^a^	–0.18^a^	0.07	0.05	0.13	0.02	- - -	- - -	- - -
14. PTY	0.46^b^	0.41^b^	0.25^b^	0.50^b^	0.30^b^	0.22^a^	0.31^b^	–0.11	0.02	0.38^b^	–0.07	0.11	0.53^b^	- - -	- - -
15. SEN	0.18^a^	0.18^a^	0.09	–0.01	0.24^b^	0.06	0.22^a^	0.14	–0.12	0.55^b^	–0.15	0.25^b^	0.20^a^	0.48^b^	- - -

Categories for strength of relationships: + 0.10 to – 0.10 = No relationship; ± 0.10 to ± 0.30 = Weak relationship; ± 0.30 to ± 0.50 = Moderate Relationship; ± 0.50 to ± 1.00 = Strong Relationship (Cohen, 1988)

^a^Significant at 0.05

^b^Significant at 0.01

## Discussion

Recent sensitization studies in the Philippines were mostly on specific-IgE profiles of Filipinos to selected grass species (pollen) and house dust mites [13, 22]. Studies on SPT using pollen from grasses and weeds have also been described [12]. After a few decades, in this study, other sources of pollen, particularly from trees were utilized for both SPT and ELISA tests. Although *M. indica*, *A. auriculiformis*, and *L. camara* are entomophilous, there have also been published reports of their allergenicity [9, 23].

Skin prick tests (SPT), which is an essential procedure to confirm sensitization in IgE-mediated allergic disease in subjects with allergic rhinitis, asthma, and atopic dermatitis, can be performed from infancy to old age [24]. Likewise, SPT is considered a safe diagnostic procedure as the occurrence of systemic reaction has decreased and cases of fatalities were extremely low [25]. Aside from time, cost and safety, SPT had a high sensitivity to aeroallergens, mainly pollen and house dust mites [26].

Factors that influence the pollen threshold values for the development of allergic symptoms are as follows: time of the season, weather conditions, pollen allergenicity, air pollution and patient characteristics [27]. The recruitment of study subjects was done near the end of the flowering season (from April to June) when most of the trees (e.g. *M. indica*, *C. nucifera*, *Eucalyptus* spp., etc.) were in full bloom and bear fruits. The flowering of some plant sources are seasonal (e.g. *M. indica*, *Eucalyptus* spp., etc.) but most flower all throughout the year (e.g. *C. nucifera*, *D. meyeniana*, *L. camara*, etc.). As previously reported [19], the concentration of airborne pollen decrease near the end of May up to the first weeks of June, due to the increasing amount of rainfall in the region. The high prevalence of allergic disease and high positive predictive value signify that there is a high probability that the amount of pollen in the air may have caused the allergic disease of the allergic subjects. Although skin tests in this study detected only almost one-third of the study subjects with allergic disease (low sensitivity), it has a high probability (specificity) that it can discriminate those who do not have an allergic disease in any of the pollen allergens used. Similarly, in another study, only 28.2% of their study subjects were sensitive to pollen allergens [8]. Allergic cases who tested negative to the skin tests require another test or another panel of pollen allergen to confirm results. Whereas, asymptomatics may develop allergic diseases later in life [28].

In this study, there’s a discrepancy between results from SPT and pollen specific-IgE (sIgE), particularly that of *M. indica*. A recent study [29] made an assumption that tropical flora produces highly glycosylated allergens that hid or mask protein epitopes. As a result, IgE binds to these non-allergenic, pollen-derived carbohydrate epitopes instead of the allergenic protein allergens. This ultimately resulted in a specific-IgE negative response. Likewise, SPT-negative subjects that are positive to specific-IgE ELISA may be due to the presence of a cross-reactive carbohydrate determinant or CCD [30–32], or the lack of pollen coat allergens, containing allergic epitopes [33]. More than 20% of allergic patients that were asymptomatic have their IgE bind to carbohydrate compounds instead of the allergen [34]. In this study, some skin test-positive subjects were asymptomatic.

Ideally, a subject that has a positive clinical history would also have positive allergen-specific test results [35]. Instead, anomalies in having either positive or negative test results occur since the cause or presence of allergic disease are not revealed in all cases with positive clinical history. Even if a patient was clinically allergic to an allergen if there were no recent exposure, then allergic symptoms may be caused by something else; or, a patient may be sensitized to an allergen but not clinically allergic to it [36]. However, a significant number of allergic sensitizations may be missed if only one type of testing was performed [37]. Thus, SPT and specific-IgE testing should not be interpreted interchangeably but instead used as complementary [38].

Among Asians, exposure to pollen in urban communities is less than in rural areas. Studies suggest that early/childhood exposure to pollen (and keeping pets) can protect against allergic sensitization up to adulthood [39]. In contrast, this protective effect of rural living had changed due to a shift in urban lifestyle in rural areas [40]. Locally, pediatric patients showed that house dust mites and cockroaches were the main allergens, followed by *Sorghum jalapense*, *A. spinosus*, and *M. indica* [41]. Likewise, *A. spinosus*, *M. indica,* together with, *Mimosa* and *A. auriculiformis* were less allergenic to children. Although *M. indica* and *A. auriculiformis* belong to < 1% of the airborne pollen in Manila [19], its role in sensitization was significant. Overall, pollen from trees in Manila showed high sensitization rates among the sample population in this study. Previous studies of plants with low pollen counts, particularly from trees, demonstrate clinical significance [42, 43]. Alternatively, in this study, trees such as *C. papaya* and *C. nucifera* belonged to > 1% of airborne pollen in Manila [19] but showed low sensitization in skin tests. This contrasts with other studies, wherein *C. papaya,* and *C. nucifera* elicited a high response to SPT [44, 45], which may be attributed to geographical location, climate and allergen exposure.

A high percentage of test subjects in this study reported a history of rhinitis and asthma, which, are allergic diseases often associated with sensitization to aeroallergens [46]. Sensitization to pollen in this study peaked at the young adult stage (20–40 years old), and male sensitization started earlier than females, as is also shown in previous studies [38, 47, 48]. A longitudinal study of an urban population in Central Italy [28] revealed a significant increase in skin prick test reactivity, using the same sample population, across age groups and particularly in subjects < 40 years old. At present, there is no accepted explanation for the sensitization shift between genders [38]. In a review assessing the impact of age on atopy, immunosenescence or a lower expression of IgE was observed as the cause of age-related decrease of positive skin test [49].

Exposure to environmental allergens increases over time as evidenced by the number of polysensitization, particularly in older age groups, as shown in this study. This polysensitization was also evident when pollen allergens were tested for cross-reactivity using an ELISA inhibition assay. Nearly all pollen, either closely or distantly related, had weak to strong associations and although of low percentage inhibition value, this may indicate the presence of cross-reactive proteins. Cross-reactivity between closely related plants reflects phylogeny, shared antigens or epitope binding sites, while cross-reactivity in distantly related plants, is due to minor allergens (e.g., profilins, lipid transfer proteins, and pathogenesis-related proteins) [50]. These minor allergens, called panallergens [51] play a significant role in the distribution of the allergic response to conserved epitopes of different allergenic sources [52].

In conclusion, pollen allergens from both arboreal and herbaceous plants used in this study yielded positive reactivities for both skin tests and specific IgE tests. Additionally, for a small community-based study population, it shows that Filipinos living in a highly urbanized city are allergenic to local pollen. Further sensitization studies should be done to assess if there would be differences between those living in urban and rural areas. Moreover, longitudinal studies comparing populations using the same tests and methods should be undertaken at different periods to have a more conclusive set of data. Likewise, an investigation of the genetic factors associated with pollen sensitization and response to therapy should be made.

## Data Availability

The datasets during and/or analysed during the current study are available from the corresponding author on reasonable request.

## Abbreviations

SPT: Skin prick test
ELISA: Enzyme-Linked Immunosorbent Assay
PTY: *Ptychosperma macarthurii*
COC: *Cocos nucifera*
MAN: *Mangifera indica*
ACA: *Acacia auriculiformis*
SEN: *Senna siamea*
LAN: *Lantana camara*
CAR: *Carica papaya*
TER: *Terminalia catappa*
EUC: *Eucalyptus* spp.
PIN: *Pinus kesiya*
DEN: *Dendrocnide meyeniana*
MIM: *Mimosa* spp.
DIC: *Dichanthium aristatum*
PIL: *Pilea microphylla*
AMA: *Amaranthus spinosus*
PBS: Phosphate buffered saline
ISAAC: International Studies of Asthma and Allergy in Children
HDM: House dust mites
SUD: *Suidaisia pontifica*
BLO: *Blomia tropicalis*
AA: Allergic asthma
AR: Allergic rhinitis
AD: Atopic dermatitis
